# Text-Based Plagiarism in Scientific Publishing: Issues, Developments and Education

**DOI:** 10.1007/s11948-012-9367-6

**Published:** 2012-04-26

**Authors:** Yongyan Li

**Affiliations:** Faculty of Education, University of Hong Kong, Pokfulam Road, Hong Kong, China

**Keywords:** Text-based plagiarism, Automated text-matching, English as an additional language (EAL) authors, Novice scientists

## Abstract

Text-based plagiarism, or copying language from sources, has recently become an issue of growing concern in scientific publishing. Use of CrossCheck (a computational text-matching tool) by journals has sometimes exposed an unexpected amount of textual similarity between submissions and databases of scholarly literature. In this paper I provide an overview of the relevant literature, to examine how journal gatekeepers perceive textual appropriation, and how automated plagiarism-screening tools have been developed to detect text matching, with the technique now available for self-check of manuscripts before submission; I also discuss issues around English as an additional language (EAL) authors and in particular EAL novices being the typical offenders of textual borrowing. The final section of the paper proposes a few educational directions to take in tackling text-based plagiarism, highlighting the roles of the publishing industry, senior authors and English for academic purposes professionals.

## Introduction

A potential distinction between the borrowing of ideas and the borrowing of text in scientific writing is suggested by such terms as *semantic reuse* vs. *textual reuse* (Collberg and Kobourov 2005), and *plagiarism of ideas* vs. *plagiarism of text* (Vessal and Habibzadeh 2007). The distinction between the two scenarios highlights a need to treat the two differently, as recently emphasized by Bouville (2008). Nevertheless, such a distinction does not justify either of the two scenarios: it is generally agreed that borrowing of ideas or else borrowing of text (with the implication that proper acknowledgement is lacking) is wrong.

Plagiarism in the sense of stealing ideas continues to be fought against as with other forms of research misconduct (e.g., fraud and duplication); meanwhile, reusing words from published papers, or “the misappropriation of language from other authors,” has recently been raised as a “quite significant trend” by some editors of scientific journals (Williams 2007, p. 2535; see also “It’s a steal,” 2007; Kara Mosher, cited in Perry 2010; Zhang 2010a). Although the papers found guilty of such practice may have “unique data results” (Kara Mosher, cited in Perry 2010), their reuse of “basic textual wording” (Kara Mosher, cited in Perry 2010) or of “eloquent phrases, sentences or even whole paragraphs” from published texts is considered by journal editors a questionable or even “morally wrong” practice (Williams 2007, p. 2535).

The primary purpose of this paper is to provide an overview of some issues and developments concerning the tackling of the phenomenon of textual borrowing, or text-based plagiarism (Li 2012a), in scientific publishing, with the hope of inspiring concerted efforts on the part of the publishing industry, senior authors, and English for Academic Purposes (EAP) professionals to educate authors, perhaps especially novice English as an Additional Language (EAL) authors, against unwarranted textual appropriation. In the following I will begin by discussing the development of automated anti-plagiarism check, and then examine gatekeepers’ stance on textual copying. This is followed by a look into the question of whether textual copying may be justifiable in some cases and a focus on EAL authors, in particular novices, as the typical culprit of textual appropriation. Then I will examine the use of pre-submission text-screening, before proposing a few areas where educational actions can be taken.

## Automated Text-Matching Screening

Automated text-matching detection in the publishing industry is a later development compared with the use of the technology in universities for checking student assignments. To trace its development in recent years, a Self-Plagiarism Detection Tool (SPlaT), produced at the University of Arizona, was applied to computer science articles to search for self-plagiarism (Collberg and Kobourov 2005); this was followed by software developed at Cornell University, which trawled papers lodged in arXiv, a preprint server that collected mostly physics papers (Sorokina et al. 2006). Out of more than 0.28 million of arXiv articles combed at the time of the study, 677 pairs of papers were found to have “at least four sentences sharing uncommon 7-grams” (common 7-grams such as *can be expressed in terms of the* were excluded) (Sorokina et al. 2006, p. 1073). These 677 pairs, or 1,086 unique articles, included cases of one paper copying from multiple sources and one source copied by more than one paper (Sorokina et al. 2006, p. 1073). Human inspection of 20 of the 677 pairs excluded four “innocent mistakes” and found three cases of almost complete duplications, with the rest showing text matching in parts of a paper, such as in the introduction section (Giles 2006, p. 524). In a later study conducted by some researchers based at the University of Texas Southwestern Medical Center, survey of the abstracts in MEDLINE (a biomedical citation index of titles and abstracts) using a search engine called eTBLAST generated a large number of potential duplicates (Errami and Garner 2008) and by October 2008, 75,000 highly similar abstracts had been identified (Butler 2008).

Meanwhile, after years of preparation, CrossRef, a non-profit membership association of publishers, joined hands with iParadigms, the software company that also produced Turnitin (educational text-matching software now widely used in universities), to launch CrossCheck, an anti-plagiarism program powered by iThenticate (iParadigms’s corporate text-matching software). Similar to the working principle of Turnitin, CrossCheck works on the basis of a continuously expanding database of scholarly full-text literature, and a text-similarity analyzer (the web-based iThenticate tool) which compares an authored work against the database. CrossCheck exposes both “very blatant unethical cases of plagiarism” as well as language reuse of various degrees of severity (Butler 2010, p. 167). The program has so far been put to test by such major publishers as Elsevier, Springer, Taylor and Francis, Wiley-Blackwell, and the Nature Publishing Group (Butler 2010; Colón 2008; Hu 2010; “Plagiarism pinioned,” 2010), and has been widely hailed as a valuable text screening tool.

To determine the nature of the offence would necessitate manual check, which was reportedly incorporated into the research using SPlaT (Collberg and Kobourov 2005), as well as the survey study of MEDLINE (Long et al. 2009) and arXiv (Sorokina et al. 2006); apparently it is now also a regular part of journals’ screening process using CrossCheck (e.g., Zhang 2010b). As a recent editorial carried in *Nature* pointed out, “plagiarism-detection software is an aid to, not a substitute for, human judgement” (“Plagiarism pinioned,” 2010, p. 160). The same editorial went on to state:

One rule of thumb used by Nature journals and others in considering an article’s degree of similarity to past articles – in particular, for small amounts of self-plagiarism in review articles – is whether the paper is otherwise of sufficient originality and interest. (“Plagiarism pinioned,” 2010, p. 160)

Statements like this issued by journals may help to settle some uncertainty among scientist authors. However, it may still be suggested that such indication of the possible use of human assessment over “originality and interest” in addition to the text-matching check does not itself provide guidance to scientists as to what levels or scenarios of textual copying are not acceptable, e.g., requiring rephrasing before peer review, or simply leading to rejection.

## Gatekeepers’ Stance on Text-Based Plagiarism^1^

Notably, the quotation above, by emphasizing the importance of using human judgement, indicates a context-sensitive approach to plagiarism. For instance, the same editorial put forward a “mitigating” scenario of language reuse that may be relevant to EAL scientists: “All plagiarism can also involve honest errors or mitigating circumstances, such as a scientist with a poor command of English paraphrasing some sentences of the introduction from similar work.”^2^ (“Plagiarism pinioned,” 2010, p. 160)

Textual copying in the introduction section of a research article does seem to be a common feature of language reuse detected in EAL authors’ papers (Brumfiel 2007; Lin et al. 2011; Sorokina et al. 2006). Paul Ginsparg, a Cornell University professor on the research team that developed software to investigate duplications in arXiv (i.e., Sorokina et al. 2006), reportedly “thinks that although such practices are ethically questionable, it is inappropriate to be overly draconian” (Brumfiel 2007, p. 8). Such a mitigating stance, of course, does not imply that textual borrowing, such as that in the introduction of an article, is positively regarded. Indeed, as cited earlier, David Williams, the editor-in-chief of *Biomaterials*, suggested that such practices are simply “morally wrong” (Williams 2007, p. 2535). Similar reservation or clear opposition to textual copying, in any section of an article, is generally expressed by editors, editorial managers, or researchers of scientific texts (see Brumfiel 2007; Butler 2010; Eckel 2010; Lin et al. 2011; Schilperoort 1995; Zhang 2010b).

The Committee on Publication Ethics (COPE), “a forum for editors and publishers of peer-reviewed journals to discuss all aspects of publication ethics” (http://www.publicationethics.org), has published two flowcharts, on *What to do if you suspect plagiarism* with *(a) Suspected plagiarism in a submitted manuscript* and *(b) Suspected plagiarism in a published article* respectively (COPE 2008). Both flowcharts distinguish between “clear plagiarism” (“unattributed use of large portions of text and/or data, presented as if they were by the plagiarist”) and “minor copying of short phrases.” The instructions over the latter (“minor copying of short phrases”) which indicate a lesser degree of severity of copying as compared with the former, are noteworthy. Specifically, within scenario (a), an arrow (indicating steps of actions to take) leads from “Minor copying of short phrases only (e.g. in discussion of research paper from non-native language speaker)//No misattribution of data” to “Contact author in neutral terms/expressing disappointment/explaining journal’s position//Ask author to rephrase copied phrases or include as direct quotations with references//Proceed with review”.^3^ And within scenario (b), an arrow leads from “Minor copying of short phrases only (e.g. in discussion of research paper)//No misattribution of data” to “Contact author in neutral terms/expressing disappointment/explaining journal’s position//Discuss publishing correction giving reference to original paper(s) if this has been omitted” and then to “Inform reader (and plagiarized author(s) if different) of journal’s actions”. It is thus clear that COPE upholds a high standard: that even “minor copying of short phrases” such as that “in discussion of research paper from non-native language speaker” is not acceptable, from the point of view of COPE, and by extension, from the point of view of the international publishers and journals that subscribe to the tenets of COPE.

In short, to journal gatekeepers, textual borrowing “in discussion of research paper” (COPE 2008) or in the introduction section (Paul Ginsparg, cited in Brumfiel 2007), despite being “mitigating circumstances” if a relatively small amount of borrowing is involved (“Plagiarism pinioned,” 2010), is not acceptable in either submissions or published papers.

## Rewriting, Rather Than Reusing, Almost Under All Circumstances

A scenario where unattributed language reuse (i.e., with a high level of similarity between two texts) seems acceptable to some journal editors, concerns the description of highly standard experimental or statistical procedures in the methods section (Lin et al. 2011, p. 5). For example, Catriona Fennell, director of journal services at Elsevier, commented: “There are only so many different ways you can describe how to run a gel” (Butler 2010, p. 167).^4^ Yet a controversial scenario is where a researcher does a series of studies, with a shared research background and following some shared, self-developed (original), experimental procedure. The researcher might thus argue that because of the parallel nature of the studies, text similarity between the separate papers in the methods section cannot be avoided (Brumfiel 2007). Changjie Hu, publishing manager at Wiley-Blackwell, has described such a case with a journal within his remit of management, and noted that after the editor of the journal had communicated with the author concerned and learned about the background for the similarity between the submitted paper and the author’s previous paper, “the editor asked the author to add the relevant explanation and references, and agreed to send the manuscript out for review” (Hu 2010). It was not clear whether the referees later approved the text similarity in the methods section in this case, but evidence of journal gatekeepers supporting this kind of textual overlap seems lacking in the literature. At the same time, publishing a series of similar articles (presumably reporting a series of similar studies) has been widely condemned as misconduct. For example, Paul M. Evans, former Vice-President of Elsevier’s Science and Technology Department in China, and currently managing director at Sage Publications Asia Pacific, talked of the need to prevent “salami” style publishing (i.e., cutting one study into thin pieces to be published separately) (Evans 2004: Evans and Huke 2011). Arnout Jacobs, manager of publishing development at Elsevier, likewise pointed out in an interview with *Keji Shibao* [Science News] in China (Wang 2008): “it is a nuisance for journal editors when researchers publish a series of highly similar papers. Often, these papers could easily be rewritten as one single excellent paper.” (cited in Zhang 2010b, p. 11).

Where entirely different studies, rather than similar studies, are reported, how about reusing methodological descriptions from one’s own previous paper, or a previous paper of one’s own research group, or someone else’s paper? Some EAL authors (including novices) seem to think this is a good strategy (as reported in Flowerdew and Li 2007; Li 2007; and Zhang 2010b). Yet again, this is not approved by experienced EAL scientist writers (Dubois 1988) or journal editors (“Plagiarism pinioned,” 2010). Using CrossCheck to screen submissions to the China-based SCI journal, *Journal of Zhejiang University*-*Science (JZUS)*, Yuehong Zhang (Journal Director of *JZUS*) found “Direct copying of Methods section, with new data inserted” to be “a particularly common phenomenon in biomedical papers” (Zhang 2010b, p. 11). She emphasized:

In principle we believe that, although much research refers to or repeats others’ successful methods in testing new materials and discussing new results, the authors should use their own language to describe and summarize their methods and ideas. (Zhang, 2010b, p. 11)

## Inexperienced EAL Authors as the Typical Culprit of Textual Borrowing

The use of computational originality-verification tools have helped to bring to the fore the typical culprit of textual copying: EAL scientists who may have difficulty in English or who have had limited experience in English writing (Brumfiel 2007; Butler 2008; “It’s a steal,” 2007; Lin et al. 2011; Sorokina et al. 2006). Defending their language reuse, some EAL scientists argued that they were “just borrowing better English” (Yilmaz 2007, p. 658) because “he or she is disinclined to sacrifice quality and accuracy for want of linguistic expertise” (Vessal and Habibzadeh 2007, p. 641). They pointed out that theirs is not plagiarism, and in scientific writing it is the reported science, not words, that count (for a reasoned discussion of the issue, see Bouville 2008). The following argument is typical (see also Zeng 2010):

Borrowing sentences in the part of a paper that simply helps to better introduce the problem should not be seen as plagiarism. Even if our introductions are not entirely original, our results are – and these are the most important part of any scientific paper. (Yilmaz, 2007, p. 658)

Speaking of textual borrowing in the introduction section, the same author pointed out this is for facilitating the publication of a paper so that their research would gain visibility (Yilmaz 2007, p. 658). Other EAL scientists who also held a textual-copying-is-harmless view suggested that “Rules of the game of scientific publishing,” being “set by native English-speakers,” “greatly disadvantage authors whose first language is not English” (Vessal and Habibzadeh 2007, p. 641); as the game is not “fair play,” reviewers and editors should “ignore such *faux pas*, since we are not sure that they would fare any better if they were to write a similar article in their own second language” (Vessal and Habibzadeh 2007, p. 641).

By contrast, yet other EAL scientists have expressed strong opposition to such laisse-faire approach to textual copying, echoing the gatekeepers’ stance discussed above. The following quote from a Chinese scientist’s blog is illustrative (see also Afifi 2007):

If you can not work out a new narration when writing a paper, your paper is perhaps limited in its academic value and contribution. A paper of real innovation is necessarily a result of someone’s word-for-word contemplation. Some friends think, since I was following a line of research in the literature, but provided new results from a different perspective, can’t I reproduce something from the literature in the introduction and literature review? Yes you can, but that way you put yourself in the position of a low-level scientist. (Yuan, 2010; my translation from Chinese).

If this Chinese scientist blogger was discussing a high versus a low standard for oneself in scientific writing which would reflect one’s attitude toward textual borrowing, indeed research has revealed that duplications (encompassing textual borrowing) tend to appear in low-profile journals (Brumfiel 2007; Long et al. 2009). To cite a contrasting example, journals published by the Nature Publishing Group have detected through CrossCheck “only trace levels of plagiarism in research articles,” “often in only the supplementary methods” (“Plagiarism pinioned,” 2010, p. 160).

Why are EAL authors often the culprit of text-based plagiarism? Some EAL authors have cited the difficulty with English and unwillingness to sacrifice the accuracy of meaning (against a need to get published for their research to gain visibility) to justify their language reuse, as noted above. Gatekeepers tend to acknowledge the language barrier as a cause, but they have also cited culture and insufficient ethics education as contributing factors (Brumfiel 2007; Butler 2008; Errami and Garner 2008; Zhang 2010a). Errami and Garner (2008), for instance, commenting on their estimate that MEDLINE holds duplications produced by scientists in China and Japan at a level well exceeding what would be proportionate to the number of the two countries’ publications in the database, suggested, “Perhaps the complexity of translation between different scripts [implying a language barrier—the present author’s note], differences in ethics training and cultural norms contribute to elevated duplication rates in these two countries” (p. 398). In addition, pressure of publication in EAL countries has also been cited as a contributing factor (Brumfiel 2007; Perry 2010; Qiu 2010).

## EAL Novice Scientists as Offenders of Textual Borrowing

Research in language education has reported differences between expert and novice writers in their attitude toward textual borrowing (Dong 1996; Flowerdew and Li 2007; Pecorari 2003). Automated screening of arXiv reveals that “while prominent (highly cited) authors are frequently victimized, they do not appear to reuse text from others” (Sorokina et al. 2006, p. 1075). Meanwhile, it is noticeable that novice/junior EAL scientists have been the key offenders in a number of reported cases of plagiarism, or extensive textual copying, if the involved novice authors’ defence that they reported original research results is accepted (see Brumfiel 2007, for a report of Turkish doctoral students of physics being charged with plagiarism, from a study of arXiv; and see Li and Xiong 1996 for description of a case involving junior Chinese scientist authors).

Participation of novice/junior EAL scientists, typically doctoral science students, in scientific publication has been widely reported (Li 2006, 2007; Blakeslee 1997; Florence and Yore 2004; Gosden 1995; Swales 1990). The involvement of novice scientists in the English publication arena has, to take the prominent case of China as an example, grown with the expansion of enrollment at the graduate (especially doctoral) level in the country’s higher education sector, and with the increasing entrenchment of English (international) publication as a graduation requirement in many universities (see Cargill et al. 2012). The graduation pressure, language barrier, confusion over what constitutes plagiarism, inadequate vigilance against plagiarism in previous and current education, and even laziness, are likely to combine to lead to a high level of textual copying in the novices’ texts. The view that “culture” has motivated plagiarism (according to some gatekeepers, as noted above) is probably not convincing in discussing the problem of plagiarism in this context, despite its apparent appeal (see also e.g., Chandrasoma et al. 2004; Li 2012a).

Where there is a lack of monitoring by experienced senior writers, textual borrowing may carry into submissions and even published papers, though CrossCheck now potentially forestalls this. A different scenario, where the senior experienced author plays a dominant role in the process of writing for publication, seems ideal insofar as eliminating textual appropriation is concerned (Li 2012b). However, in such cases how much a novice learns in terms of the need and strategies for such elimination, is questionable, because the process of eradicating the novice’s textual copying in his/her preliminary text is actually embedded in the reiterative rhetorical construction of a paper, in which the novice has had little participation (Li 2012b).

## Pre-Submission Text-Screening for Eliminating Textual Borrowing

A recent initiative by iParadigms, the company that provided software technology for both Turnitin and CrossCheck, is to extend the iThenticate solution (the text-matching software that CrossCheck relies on) to serve individual authors and researchers (research.ithenticate.com). The idea is to allow individuals to screen their work against a massive live database of scholarly literature before submission (“iThenticate introduces,” 2011). “Within seconds, iThenticate produces a report that highlights content matches and provides links to significant text found within iThenticate’s databases.” (“iThenticate introduces,” 2011)

While the value of this new initiative seems obvious, a potential concern, as teachers may have by allowing students access to Turnitin (Emerson 2008), may be: does individual authors’ access to such tools facilitate “smarter plagiarism” (“Use of anti-plagiarism software,” 2011)? This concern was expressed by Geoffrey Bilder, director of strategic initiatives at CrossRef, when CrossCheck was being piloted in 2007 before its formal launching in 2008. Projecting the prospect of authors running an automated check of their papers prior to submission, the director said this would give honest authors a chance to root out inadvertent verbatim cut and paste; yet the downside could be “It might just force people to become more sophisticated plagiarists” (cited in Butler 2007, p. 633). Such smart plagiarism will pose a challenge to automated detection. Indeed, in a *Nature* report featuring the technology that searched through arXiv for duplications (Sorokina et al. 2006), it was noted that “the software is unable to pick up ‘intelligent plagiarism’, where material copied from another author is reworded.” (Giles 2006, p. 525) From the point of view of helping authors to get rid of inadvertent, relatively minor extent of textual borrowing in a manuscript, this pre-submission text-screening scheme will certainly be valuable. Yet ill-intentioned cheating by re-wording long stretches of text (i.e., without proper attribution of the sources) to circumvent detection remains likely.

Both pre-submission automated checking by the authors themselves and post-submission vetting by the journals are ad hoc mechanisms, in the sense that they are about checking a paper after it has been written. Even so, the current (still growing) integration of originality-verifying software into the scholarly publication process is sure to alert research writers to the issue of plagiarism, including text-based plagiarism, so that they are more vigilant *during* the composing process.

## Addressing Text-Based Plagiarism Through Education

In the foregoing sections I reviewed issues and developments concerning the occurrence and handling of text-based plagiarism in scientific publication. In this section I will make proposals with an educational intent, highlighting the roles of three parties: the publishing industry, senior authors (by which I refer in particular to disciplinary advisors at the graduate level), and EAP professionals.

### An Explicit Guide Teaching About Text-Based Plagiarism Is Needed in the Scientific Publishing Industry

Scientific journals have constantly maintained a firm policy against plagiarism, as can be seen in the numerous editorials carried in a wide range of journals and conveying a message of zero-tolerance on plagiarism. However, distinction between the stealing of science and the copying of language, as explicitly stated in editorials or by individual editors, has been quite recent. The foregoing sections of the present paper have shown that text-based plagiarism is a valid notion in the world of scientific publication and it is an issue worthy of wider discussion. In addition to the use of CrossCheck, the publishing industry—COPE, publishers, and individual journals—should perhaps provide on the Web a downloadable handbook, which spells out guidelines dedicated to illuminating and exemplifying what is text-based plagiarism, the forms it takes, and how to avoid it in varied scenarios. It can be noted that scientific academia has no lack of handbooks on ethics, such as *On being a scientist: Responsible conduct in research* (Committee on Science, Engineering, and Public Policy, National Academy of Sciences, National Academy of Engineering, and Institute of Medicine 1995), and *Publishing ethics resource kit* (n.d.), just to name two. Yet it seems text-based plagiarism needs to be separately addressed, in the level of detail and depth it deserves, for dispelling various misconceptions that scientist authors (especially novices) may have concerning language reuse, and for providing a valuable reference in the ethics education and English enhancement training for these authors. It is worth noting that an in-depth analysis of text similarity in scientific writing is in the plan of a team of recent recipients (and the first Chinese recipients) of the COPE grant for publication ethics research. Yuehong Zhang, who leads the China-based SCI publication *Journal of Zhejiang University*-*Science* (noted earlier), with her editorial research team, has won COPE’s December 2010 grant, to work on their project *CrossCheck guidance: An analysis of typical cases of plagiarism in different disciplines*. A major target of the project is to produce a handbook listing the “typical cases of plagiarism in different disciplines” based on investigations using CrossCheck (“COPE grant awarded,” 2011, p. 3). Such a handbook is likely to become a profoundly valuable reference for scientific writing and publishing.

Where a journal has a mentorship scheme supporting EAL or inexperienced authors (e.g., Mišak et al. 2005) (though this seems extremely rare), even with the absence of a comprehensive guide geared to teaching about text-based plagiarism, some illustrative guidance should still be a valuable component of the mentoring mechanism.

### Senior Authors Should Take Responsibility in Educating Novices

David Williams, editor-in-chief of *Biomaterials* quoted earlier, on observing “in several of the cases [of text-based plagiarism] that have come to light, these authors [senior authors] have graciously admitted that they did not diligently read the final version that was submitted,” proposed that “the ultimate responsibility does lie with the senior authors” (Williams 2007, p. 2535). Similarly, the *Nature* editorial, also cited above, ends by stating the following: “It is crucial that research organizations in all countries, and particularly the mentors of young researchers, instil in their scientists the accepted norms of the international scientific community when it comes to plagiarism and publication ethics.” (“Plagiarism pinioned,” 2010, p. 160) In addition, the flowcharts of COPE, excerpted above, clearly indicate the responsibility of senior authors in helping to guard against unwarranted language reuse in submissions.

Senior authors’ awareness of the issue will be reflected in their teaching of novices. Web-based, easily accessible, and user-friendly materials, such as the COPE flowcharts (and promisingly the handbook that Yuehong Zhang’s research group aims to produce in the near future), are potentially valuable teaching materials, both for supervisors’ self-education (see Hamp-Lyons 2011) and for mentoring novices against text-based plagiarism. At the end of a previous section of this paper (“EAL Novice Scientists as Offenders of Textual Borrowing”), I referred to a scenario where a senior experienced author may play a dominant role in the writing for publication process, by rewriting novices’ initial drafts of research papers; I suggested that the extent of novices’ learning regarding the eradication of textual copying may be limited, with their lack of substantial participation in the rhetorical construction process of a paper (see also Li 2012b). Although it is likely that such division of labor between a supervisor and novices is effective in a local context, by facilitating a research group’s efficient publication of papers, it does also seem to depend on the novices as well as the supervisor to make a difference, without diminishing the research group’s track record of publication, to enhance the novices’ participation in the process of writing for publication (as for instance, described in Florence and Yore 2004 and Li 2006). Disciplinary supervisors’ conscientious effort to engage novices in a program of hands-on writing practice whereby they receive enough exposure to and have sufficient practice in research writing will be crucial for facilitating the novices’ learning to write without text-based plagiarism.

### English for Academic Purposes Professionals May Utilize a Wide Range of Teaching Resources

The issue of text-based plagiarism in scientific publishing intimately bears upon the stakeholders that EAP professionals commonly work with, i.e., authors who use English as an additional language, including novice EAL scientists. EAP practitioners, perhaps especially those working with EAL students, have extensively addressed the issue of how to help novices to avoid text-based plagiarism in disciplinary writing (e.g., Abasi and Akbari 2008; Barks and Watts 2001; Krishnan and Kathpalia 2002; Tardy 2009), more recently with particular recourse to specialized corpora of disciplinary texts which have facilitated educating EAL novices on learning to use recurrent language (e.g., Bianchi and Pazzaglia 2007; Lee and Swales 2006; Simpson-Vlach and Ellis 2010). In addition to incorporating the immensely valuable corpus-based strategies, a wide range of resources can also be integrated into the EAP instruction. These include, firstly, relevant views of gatekeepers and supervisors (as found, for instance, in Dong 1996; Li 2012b; Williams 2007), and secondly, currently available user-friendly resources such as the COPE flowcharts and relevant sections in *On being a scientist: Responsible conduct in research*, and *Publishing ethics resource kit* (both noted above). In addition, a case study approach centering around discussions of publicized cases of plagiarism, especially where the accused authors argued that they had reported original research (such as the cases reported in Brumfiel 2007; Li and Xiong 1996), seems potentially productive (Myers 1998). Furthermore, recent developments in the use of CrossCheck by journals and the possibility of a pre-submission check can be explored with their learning implications pursued. With EAL students, the influence of previous education and the native culture can be discussed and debated; and where the teaching takes place in an English as a Foreign Language (EFL) context, local Web-based discussions of the issue of plagiarism, reports of plagiarism cases in the media, and ethical codes for research conduct issued at local universities, all make valuable teaching resources. Apart from engaging novices in activities that may be developed from using these open resources, guided examination by students of their own texts and their supervisors’ revisions, as well as of source use in journal articles (and for possible comparative findings, using low-profile and high-profile journals), can create an enlightening learning experience. By raising students’ rhetorical awareness about writing from sources, EAP practitioners will also be indirectly facilitating novices’ participation in the process of writing for publication, which in turn is likely to strengthen their ability to produce texts unique to the presentation of their own research.

In short, the publishing industry, senior authors, and EAP professionals need to collaborate in the educational undertaking of addressing the problem of text-based plagiarism and promoting healthy scholarly publishing.

## Footnotes

1 Here “gatekeepers” is used to generically refer to the personalities and organizations in the publishing industry that are involved in laying down protocols and assessing the merits of a manuscript on its course of seeking publication.

2 The word “paraphrase” in this quote does not seem to be in the sense of the word as commonly understood, for instance, by EAP professionals. Instead, the word here seems to be equivalent to *patchwriting* (Howard 1995).

3 It should be noted that to “include as direct quotation with references” is not a common practice in science and engineering research texts, compared with the case in humanities and social science texts.

4 However, some would point out that such standard procedures can just be indicated with a citation rather than being repeated (Swales 1990, pp. 166–167).
